# Variations in time and space of an Andean wild population of *T. infestans* at a microgeographic scale

**DOI:** 10.1186/1756-3305-7-164

**Published:** 2014-04-03

**Authors:** Philippe Brémond, Renata Salas, Etienne Waleckx, Rosio Buitrago, Claudia Aliaga, Christian Barnabé, Stéphanie Depickère, Olivier Dangles, Simone Frédérique Brenière

**Affiliations:** 1 IRD, Institut de Recherche pour le Développement, UMR INTERTRYP (IRD-CIRAD), Interactions hôtes-vecteurs-parasites dans les infections par trypanosomatidae), 911 Av. Agropolis, 34394 Montpellier, cédex 5, France; 2 Instituto Nacional de Laboratorios de Salud (INLASA), Laboratorio de Entomología Médica, 14 Rafael Zubieta #1889, Miraflores, Casilla M-10019 La Paz, Bolivia; 3 IRD, Institut de Recherche pour le Développement, UR 072, Laboratoire Evolution, Génomes et Spéciation, UPR 9034, Centre National de la Recherche Scientifique (CNRS), 91198 Gif-sur-Yvette CedexFrance et Université -Sud 11, Paris 91405, Orsay Cédex, France; 4 Instituto de Ecología, Universidad Mayor San Andrés, Cota Cota, La Paz, Bolivia

**Keywords:** Wild *Triatoma infestans*, Bolivia, Capture-mark-recapture, Temporal variation, Displacement

## Abstract

**Background:**

Wild populations of *Triatoma infestans* are now believed to be the source of reinfestation of dwellings in some Andean areas and could impede the full achievement of vector control campaigns in this region. Given the poor knowledge of these populations in natural conditions, their basic biology traits, such as monthly demographic variations and movements of individuals, were explored.

**Methods:**

A previously identified wild population of *T. infestans* in a field adjacent to a group of isolated houses in an Andean valley (department of La Paz, Bolivia) was explored using regular capture assays over 13 months in 50 sites selected at the beginning of the study. The capture-mark-recapture method was applied monthly using mouse-baited adhesive traps for captures and fingernail polish of different colors for the marking.

**Results:**

The monthly capture assays did not show significant differences between rainy and dry seasons, showing evidence for a certain stability of the wild *T. infestans* population with only the nymph population tending to decline during the middle of the rainy season when rain is more intensive. Throughout the study, the monthly average number of bugs was 51.1 ± 25.3 per assay, 91.1% were nymphs, and they were found at 30 of the 50 sites (60%). The number of times a site was positive varied from one to 13. Site infestation was associated with the underground position of the traps, and rocks around and in the surroundings of the traps. The recaptures after marking were successful (138 recaptures over the study). The marking made it possible to detect for 14.5% of the recaptures significant movements of adults (up to 168 m) and nymphs (up to 34 m). Some bugs (nymphs and females) were recaptured after 5 months. For adults, recaptures (46 in total) mostly occurred between September and March. Females were recaptured twice as frequently as males.

**Conclusion:**

The Andean wild populations of *T. infestans* showed a strong spatial and temporal stability during the year-long study. Dispersal may occur mainly during the rainy season. The capture-mark-recapture method was successful and the longevity of the bugs and the distances covered by nymphs and adults were recorded.

## Background

Chagas disease remains a major public health problem in Latin America with 8–9 million people infected with the causative agent, *Trypanosoma cruzi*[1]. *Triatoma infestans* (Reduviidae, Triatominae) is the main vector of Chagas disease in most countries of South America. As a result of the Southern Cone Initiative to control and eliminate Chagas disease (INCOSUR), several countries have certified the interruption of transmission, including the vector-borne mode, throughout their territory for several years (Brazil, Chile and Uruguay). Argentina, Paraguay, and Bolivia have achieved interruption of transmission in several of their provinces or departments but reinfestation still occurs in others [2,3]. Control programs were conducted against domestic populations of *T. infestans* using residual insecticide spraying of the human dwellings without taking into account wild foci of *T. infestans* although a few wild populations were initially known only in the Cochabamba valley in Bolivia [4,5].

More recent findings of wild populations of *T. infestans*, in different Andean valleys in Bolivia [6-8] and in non-Andean regions of Argentina, Bolivia, Chile, and Paraguay [9-13] indicate that wild populations of *T. infestans* have a wider geographic distribution than previously thought. The ability of these populations to colonize dwellings is still not well understood, but without appropriate vigilance methods, they may well be able to colonize peridomicile areas and then dwellings [8,14-16]. Concerning the department of La Paz in Bolivia, where the current study was conducted, the INCOSUR certified the interruption of the vector transmission by *T. infestans* in 2011, (XVIIIa. reunión de la comisión Intergubernamental de la Iniciativa Subregional Cono Sur, Cochabamba, Bolivia, 27–29 de Julio, 2011). At the same time, we reported the discovery of highly infected wild populations of *T. infestans* in many sites in two valleys of this department [8]. In this context, the study of the biological characteristics and the movements of these wild populations in their natural habitat is essential to understand their basic biology, demography, and ethology.

Taking as the study case a semi-anthropized area on a mountainside close to isolated houses in a valley of the La Paz department, regular trappings of wild *T. infestans* were performed during over a 1-year period using the capture-mark-recapture method to assess seasonal variations of the population and the movements of the insects. The current seasonal fluctuations of wild *T. infestans* were not highly pronounced since the population was maintained over the year. The capture-mark-recapture method detected several displacements of nymphs and adults and the distances that they had covered.

## Methods

### Study area

**Figure 1:**
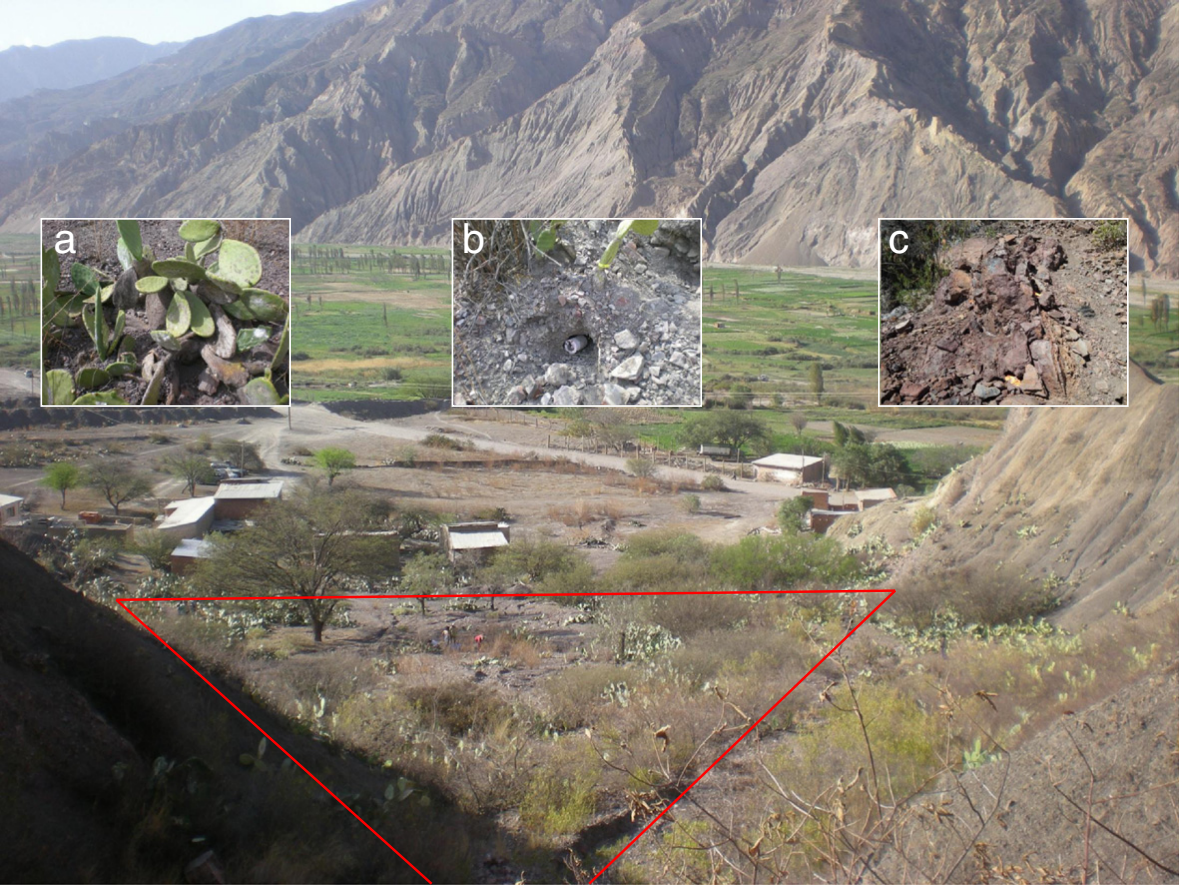
**Area of capture.** The red frame globally surrounds the area where the traps were placed. The small pictures are main ecotopes were the *T. infestans* were found: **a)** foot of prickly pear, **b)** small burrow, **c)** scree of small and median rocks.

The area consists of an abandoned field of prickly pears and cactus, adjacent to some isolated houses outside the village of Huayhuasi (Figure 1). This area is flanked by high mountains and located in the Rio La Paz valley (16°42′46.91″S; 67°59′27.13″W, 2762 m) 27.6 km from the city of La Paz. This valley has been under vector control performed by the National Chagas Disease Program (PNCH) since 2003, but the area is still suffering from sporadic domiciliary reinfestation by *T. infestans* despite insecticide spraying. A preliminary study showed that the field was colonized by wild populations of *T. infestans*[8]. The climate in the Rio La Paz valley is semi-arid and according to the monthly variations of maximal and minimal temperatures and precipitations two main seasons can be distinguished: the rainy season (November to April) and the dry season (May to October) with annual variations. Data corresponding to the study period were downloaded from the national recording system data (http://www.senamhi.gob.bo/) for the closest station (Mecapaca, 16°40′16″S; 68°01′06″W, 2680 m) to list the temperatures and precipitation levels for each day of trapping.

At the beginning of the study, 50 capture sites were chosen in the study field, in such a way as to distribute them over the area taking into account that they can be habitats for triatomines (small burrows, shelter under stones, cactus, and prickly pear) in which the traps could be hidden and sheltered from the sun. The sites were distributed in an area of around 7320 m^2^. The lower part of the land is slightly sloping and has many prickly pear and cactus plants, and several burrows of small animals were observed. The upper trapping area is a steep scree where eight sites were selected (sites 23–30). Each site was geo-referenced. In each capture site, one trap was located. The precise characteristics of each site and the surrounding area (circle of about 1 m radius) were reported. This report aimed primarily to detail the architecture and material where the traps were set, and the mineral and vegetal cover in the immediate environment of the traps.

### Trapping design

Bugs were caught using mice-baited adhesive traps [17]. They were set in the afternoon and inspected the next morning. The sex and stages of the bugs collected with the traps were determined in the field according to morphological criteria [18]. Then all insects were gently detached from the traps and released at the same site of capture after being marked except for first- and second-instar nymphs. The bugs were labeled using a specific color or a combination of two different colors of fingernail polish for each trapping assay. By staining different locations on the thorax of the adults and staining different legs of the nymphs for each site during the same assay, the adults were individually identified while the nymphs captured during a single assay at the same site were identically marked because they were too numerous to apply individual markings. Trapping assays were performed monthly from August 2009 to August 2010 (13 months) to analyze the spatiotemporal dynamics of the population. Nevertheless, additional assays were performed each week at the beginning of the study to 30 September 2009, and then every 2 weeks until 2 March 2010 to explore the movements of bugs more precisely.

### Mapping and spatial analysis

After having geo-referenced each capture site, an information system was incremented using the SavGIS (freeware developed by the IRD) programs to link the database of the field results obtained at each site. The distributions of the sampling sites (50 selected at the beginning of the study) and those positive for nymphs (they can be equated to natural colonies of *T. infestans*) were analyzed using the coordinates of each point and the vertex of the determined quadrat, by means of the nearest neighbor distance function G(r) [19] in the Spatstat package of R software “Development Core Team” [20]. This function represents the correlation of the cumulative function G of the r distance from a random point to the closest point. Following Baddeley *et al.*[21], we also used three different estimators of G(r) to take into account the influence of the edge of the sampling area: “rs”, the reduced sample estimator, “km”, the three-dimensional version of the Kaplan-Meier estimator, and “Hanisch”, the three-dimensional generalization of the ”Hanisch” estimator. The distribution pattern (random, aggregated, or regular) of the points was evaluated by comparing the data observed with the null model of complete spatial randomness [19]. We computed upper and lower 95% confidence intervals (CI) using 1000 simulations to evaluate the spatial distribution pattern.

## Results

### Monthly captures of *T. infestans*

**Figure 2:**
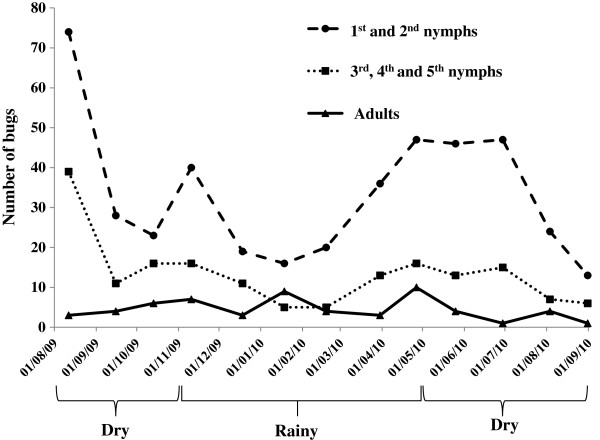
**Total number of bugs (nymphs and adults) collected over the study period.** Each point is the sum of bugs collected at the 50 study sites during each assay.

During the 13 monthly assays, a total of 650 traps were set in the 50 selected sites; 22.1% (144) of them were positive with at least one specimen caught. A total of 665 *T. infestans* specimens (marked and unmarked) were captured with an average of 51.1 ± 25.3 bugs per assay (Table 1, Figure 2). Most of the bugs were young nymphs; the total number of first- and second-instar nymphs reached 65.1% and adults were only 8.9%, with a male-to-female ratio of 0.55, significantly different from unity (chi-square test with fixed probability, *P* < 0.01). The numbers of positive traps and collected bugs varied over the months but were not significantly different between the rainy and dry seasons: 22% of the traps were positive in both seasons (*X*^2^ = 0.007, d. f. = 1, *P* > 0.05), the number of adults was 6.0 ± 3.1 vs. 3.3 ± 1.8 per month, respectively (Mann–Whitney U = 11.5, *P* > 0.05), and the number of first- to fifth-instar nymphs was 40.7 ± 17.6 vs. 51.7 ± 31.0 per month, respectively (Mann–Whitney U = 17, *P* > 0.05). However, nymph populations tended to decline during the middle of the rainy season (January and February), when the rains are more intensive. Moreover, the rate of positive traps was not significantly correlated with the minimum and maximum temperatures recorded during the day of each assay (Mecapaca station) or to the level of monthly precipitations (Spearman nonparametric test of correlation, *P* > 0.05); however, the number of captured bugs was inversely correlated to the minimum temperatures corresponding to the capture day (Spearman correlation coefficient = 0.58, *P* < 0.05).

**Table 1:** **Monthly captures of wild *T. infestans *** **and climatic conditions over 1 year**

**Assay code**	**Assay date**	**Daily minimum temp.**^**a**^	**Daily maximum temp.**^**a**^	**Precipitation (mm)**^**b**^	**Season**	**No. of positive traps**	**% positive traps**^**c**^	**No. of *T. infestans***	**No. of *T. infestans *** **per positive trap**
C1	11/08/2009	4.6	23.2	6.2	Dry	15	30.0	116	7.7 ± 9.1
C6	15/09/2009	7.3	22.6	29.7	Dry	9	18.0	43	4.8 ± 2.4
C9	13/10/2009	8.7	24.2	19.6	Dry	12	24.0	45	3.7 ± 3.3
C11	10/11/2009	11.3	24.9	18.9	Rainy	16	32.0	63	3.9 ± 3.6
C14	18/12/2009	10.6	23.7	69.7	Rainy	8	16.6	33	4.1 ± 3.2
C16	18/01/2010	11.6	24	58.2	Rainy	12	24.0	30	2.5 ± 2.0
C18	18/02/2010	11.8	24.7	91.4	Rainy	7	14.0	29	4.1 ± 3.3
C20	30/03/2010	11.4	24.8	15.4	Rainy	11	22.0	52	4.7 ± 4.5
C21	26/04/2010	9.2	24	1.5	Rainy	12	24.0	73	6.1 ± 10.7
C22	25/05/2010	7.1	22.6	7.2	Dry	8	16.0	63	7.9 ± 8.3
C23	29/06/2010	5.4	22.4	0.0	Dry	11	22.0	63	5.7 ± 6.5
C24	03/08/2010	3.9	23.2	2.5	Dry	16	32.0	35	2.2 ± 1.6
C25	31/08/2010	5.5	23.2	5.8	Dry	7	14.0	20	2.8 ± 1.9
					Total	144	22.1	665	4.6 ± 5.7

^a^The minimum and maximum temperatures corresponded to the average of the daily temperatures recorded during each month at the Mecapaca station; ^b^Total precipitation during the corresponding month (for C24 July recordings were used; ^c^The total number of traps per assay was 50.

### Triatomine captures per tracking site

**Figure 3:**
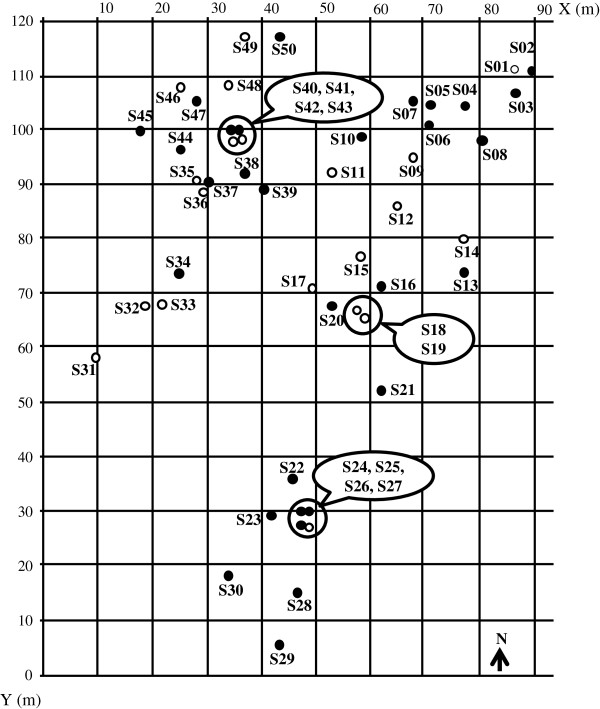
**Spatial distribution of the sites positive for wild *T. infestans*(black circle) and negative sites (white circle) during the 13 monthly assays.** The circled sites are those very close each other (less than 60 cm).

Throughout the monthly trappings, specimens of *T. infestans* were caught at least once in 30 of the 50 sites (60%). The spatial distributions of the positive and negative sites over the study period are shown in Figure 3. For nymphs and adults separately, these distributions were quite similar; however, in nine positive sites no adults were captured and in five sites only adults were captured (S07, S16, S20, S34, and S37). The number of times a site was positive varied from one to 13 (i.e., every trapping assay positive). Eleven sites were positive only once, and two were positive at each assay.

### Spatial analysis

**Figure 4:**
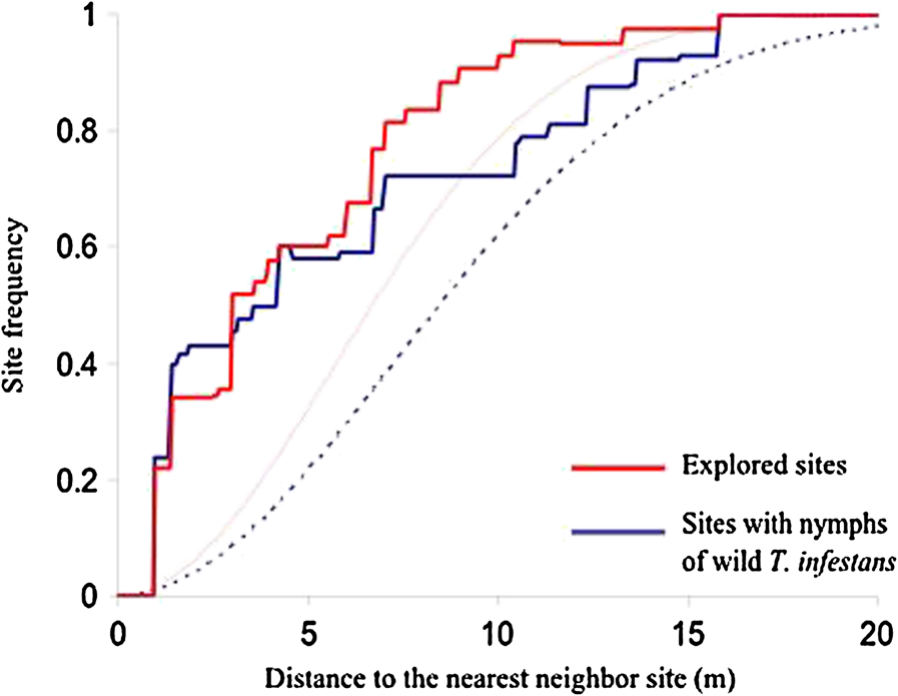
**Values of the nearest neighbor distance function G(r) calculated using the reduced sample estimator related to the distance between sampling sites with T. infestans nymphs (blue) and all explored sites (red) at the study area.** Dashed lines represent the null model of spatial randomness for both curves.

The spatial analysis of the 50 sites selected at the beginning of the study (potential habitats of *T. infestans*) showed an aggregated distribution for the majority of them (80%), for distances less than 10 m; indeed, in Figure 4 we observed that the corresponding curve (plain red line) is above the one calculated for random distribution (dashed red line); it is a clustered pattern when the curve is above the random one and dispersed when it is below. Similarly, we analyzed how the positive sites were distributed with nymphs (at least one nymph during the monthly follow-up) among the 50 sites. A nearly identical aggregation pattern was observed with aggregation distances slightly increased compared to the aggregation pattern of all the 50 sites (up to 15 m). The application of corrected indexes that take into account the influence of the edge of the sampling area did not modify the results of the two analyses.

### Microenvironment and positivity of captures

At each capture site, the traps were set on the ground or underground between stones, earth, or plants. Table 2 presents the microenvironmental descriptive variables and their association with the *T. infestans* infestation of the sites. Rates and frequencies of site infestations as well as abundance of bugs at the sites were associated with the underground position of the traps, and theses indices were higher when the traps were between rocks. The presence of rocks in the surroundings of the traps was also associated with larger colonies and higher infestation frequency. As expected, traps set in burrows were significantly more infested with higher colonies of bugs than traps set in other habitats. Also, sites where animal feces were observed (three cases) were infested more frequently with significantly larger colonies.

**Table 2:** **Features of the sites associated with *T. infestans *** **infestation**

**Descriptive variable**	**Number of sites**	**Percentages of sites infested with *T. infestans***^**a**^	**Average crowding index of *T. infestans***^**b**^	**Average number of reiterated infestations during the trial period**^**c**^
Location of the trap				
On the ground	26	46.1 (12)	5.9 ± 24.8	1.1 ± 2.0
Underground	24	75.0 (18)	21.3 ± 29.3	4.8 ± 4.8
*P*-value		**0.038**	**0.002**	**0.002**
Material where the trap was set				
Rocks	12	75.0 (9)	28.9 ± 36.1	6.5 ± 5.2
Earth	15	60.0 (9)	8.3 ± 15.4	2.1 ± 3.1
Vegetation	19	47.4 (9)	7.7 ± 28.9	1.2 ± 2.2
Mixed	4	75.0 (3)	11.5 ± 21.7	3.0 ± 4.7
*P*-value		0.43	0.17 ^d^	**0.002**^**d**^
Burrow				
Yes	13	84.6 (11)	24.6 ± 34.1	4.7 ± 5.0
No	37	51.3 (19)	9.3 ± 24.7	2.2 ± 3.5
*P*-value		**0.035**	**0.014**	**0.032**
Presence of animal feces at the site				
Yes	3	100 (3)	67.7 ± 39.5	11.3 ± 2.1
No	47	57.4 (27)	9.8 ± 23.5	2.3 ± 3.5
*P*-value		0.26^e^	**0.008**	**0.007**
Presence of stones around the trap				
Yes	14	71.4 (10)	27.8 ± 34.6	6.2 ± 5.2
No	36	55.5 (20)	7.7 ± 23.0	1.6 ± 2.5
*P*-value		0.30	**0.027**	**0.011**
Presence of earth around the trap				
Yes	41	58.5 (22)	10.8 ± 27.0	2.1 ± 3.4
No	9	100 (8)	24.5 ± 30.7	6.3 ± 5.1
*P*-value		**0.051**	**0.034**	**0.012**
Presence of vegetation around the trap				
Yes	24	54.2 (12)	8.1 ± 26.8	1.5 ± 2.7
No	26	76.9 (18)	18.1 ± 28.5	4.1 ± 4.7
*P*-value		0.16	**0.029**	**0.037**

^a^The *P*-value was evaluated with the Chi 2 test (significant *P*-value in bold); ^b^The crowding index is the average number of triatomines per site and the P-value was evaluated with the nonparametric Mann–Whitney test; ^c^the P-value was evaluated with the nonparametric Mann–Whitney test; ^d^AMOVA variance analysis was applied; ^e^Because of the sample size, the Fisher exact test was applied; the significant values are in bold.

### Capture-mark-recapture

**Figure 5:**
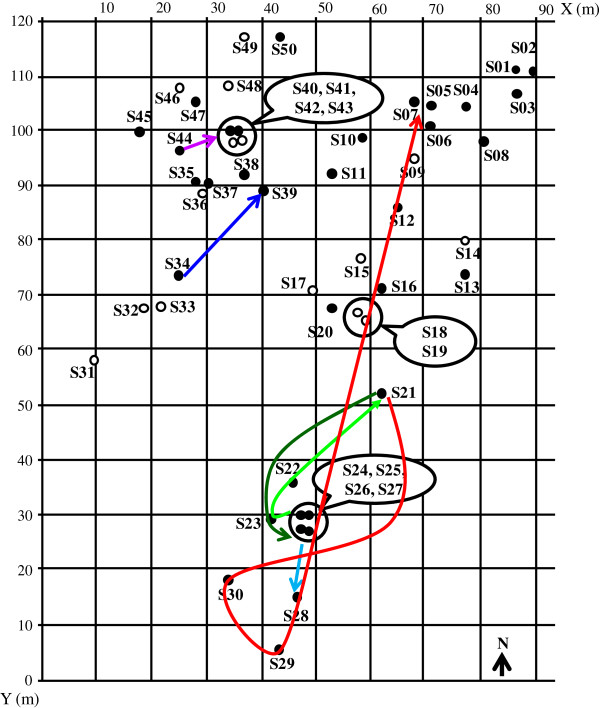
**Detail of the observed displacements of six females during the 13 monthly assays (colored lines).** The circled sites are those very close each other (less than 60 cm).

Monthly and additional assays were joined for this analysis, with a total of 1146 *T. infestans* captured (marked or unmarked) during the 25 assays. Table 3 summarizes the total number of marked specimens and the results of the recaptures. Over the duration of the study, a total of 301 *T. infestans* (third-, fourth-, and fifth-instar nymphs, males and females) were marked and 138 recaptures were obtained. In most cases (118/138, 85.5%), bugs were recaptured at the same site where they were previously captured or at nearby sites (< 60 cm), corresponding to the same block of stone or to very close burrows that probably communicate. The other recaptures (20/138, 14.5%) corresponded to bugs that moved significantly from one site to another between two assays. For the seven recaptures of nymphs, the distances covered presented a wide range of several meters: 13 m for the third-instar nymph, 6 m and 24 m for the two fourth-instar nymphs, and 10 and 34 m for the three fifth-instar nymphs. The 33 recaptures of females corresponded to 12 marked females that were recaptured one to seven times, and the 13 recaptures of males corresponded to ten marked specimens that were recaptured once or twice (Table 4). There was no significant difference of recapture proportion between males (10/28) and females (12/40) (*X*^2^ = 0.25, d. f. 1, *P* > 0.05), but females were recaptured twice as frequently as males (2.75 ± 1.86 vs. 1.3 ± 0.48) (Mann–Whitney U = 29, *P* < 0.05). The proportion of recaptures after significant displacement was 11 out of 33 (33.3%) for females and two out of 13 (15.4%) for males (*X*^2^ = 1.48, d. f. 1, *P* > 0.05). Six females were recaptured one or more times at the same site or sites that were very close to the same stone block (sites 24–27). It can be noted that six other females moved during the year browsing a maximum of 168 m. The trails of each one are illustrated in Figure 5. Five males were recaptured at the same site, three others had moved in the same stone block (sites 24–27) and two others moved 26 m and 64 m. Another very different point between males and females is that some females were recaptured after many more days post-marking than males; however, the averages were not significantly different between sexes: 63.0 ± 72.0 and 25.9 ± 15.3 days, respectively (Mann–Whitney U = 160, *P* > 0.05). Of the 138 recaptures, 80 (60%) were obtained within 1 month after marking. More remarkable was the recapture of five insects that had been marked more than 5 months before (> 151 days), two fourth- and one third-instar nymph, and one female recaptured after 196 and 318 days and another female after 231 days. For both sexes, recaptures (46 in total) occurred between September and March except for three females recaptured during August 2009 and 2010.

**Table 3:** **Capture-mark-recaptures of nymphs and adults of wild *T. infestans***

	**Stage of specimens**					
	**3**^**rd **^**instar nymph**	**4**^**th **^**instar nymph**	**5**^**th **^**instar nymph**	**Male**	**Female**	**Total**
No. of marked specimens	93	67	73	28	40	301
No. of recaptures	32	30	30	13	33	138
No. of recaptures at the same site or sites very close (< 60 cm)	31	27	27	11	22	118
No. of recaptures at a different site	1	3	3	2	11	20

**Table 4:** **Details of recaptures of *T. infestans *** **adult specimens**

	**No. of recaptures**	**No. of recaptures in the same site**	**No. of recaptures at a different site**	**Codes of visited sites**^**a**^	**Number of days after the marking for the successive recaptures**	**Total covered distance (m)**^**b**^
Female code						
1	1	1	0	S24	7	0
2	1	1	0	S44	7	0
3	1	1	0	S24, S27	13	0
4	1	0	1	S34, S39	231	22
5	2	1	1	S44, S43	14, 29	9
6	2	1	1	S24, S28	55, 69	15
7	3	0	3	S24, S23, S22, S21	7, 14, 28	35
8	3	3	0	S21	21, 28, 35	0
9	3	3	0	S26, S25, S27	53, 55, 69	0
10	4	4	0	S24, S25, S27, S24	7, 14, 21, 34	0
11	5	3	2	S21, S23, S24	13, 28, 56, 122, 140	36
12	7	4	3	S21, S30, S29, S27, S07	7, 70, 84, 111, 125, 196, 318	159
Male code						
1	1	1	0	S13	7	0
2	1	1	0	S21	10	0
3	1	1	0	S26, S25	14	0
4	1	0	1	S21, S25	14	26
5	1	0	1	S21, S02	21	64
6	1	1	0	S21	22	0
7	1	1	0	S43	59	0
8	2	2	0	S25,S27	14, 28	0
9	2	2	0	S43	31, 43	0
10	2	2	0	S24, S25, S24	31, 43	0

^a^The numbers of the sites referred to Figure 2; ^b^The distance covered was equated to zero between the following sites: S24 to S27 (same block of stone), S40 to S43 (burrows less than 60 cm away), and S18-S19 (foot of cactus less than 60 cm away).

## Discussion

Since the discovery of an increasing number of wild *T. infestans* populations, the epidemiological risk they pose for human health is a central issue for the control of Chagas disease transmission. It is therefore important to study these wild populations in their natural environment. In the department of La Paz, it was very surprising to find abundant wild populations of *T. infestans* while the National Chagas Disease Program (PNCH) had declared the interruption of transmission following a comprehensive evaluation of dwelling infestation that had decreased to less than 3%. Moreover, in recent years, during revisits of several Andean wild foci, we observed their persistent positivity generally at similar rates (data not shown). Also, we decided to study a wild population of *T. infestans* over 1 year and to examine time and space variations of triatomines and their displacements.

### Seasonal variations

During the year, nymph and adult populations fluctuated, but the trends were not highly pronounced: the curves showed a decline of the young and older nymph populations during the middle of the rainy season, when the rains were more intensive, and an increase when the temperatures were the highest (October, November, March, April). The only other focus of wild *T. infestans* that is documented is Cotapachi, a site a few kilometers from the city of Cochabamba (400 km from La Paz city) in the Cochabamba valley where the first wild foci were discovered. In this area, a significantly greater abundance of first- to fourth-instar nymph populations was observed during the rainy season compared to the dry season [7]; the authors also characterized this population by only one occurrence of nymph emergence per year, as observed in central Argentina [22]. In the present study, the emergence of nymphs appeared to occur twice a year, corresponding to milder weather, before the rains and before the cold weather, giving rise to overlapping generations. Temperature and humidity are certainly one of the factors that modulate nymph abundance and emergence.

In addition, microclimate conditions are closely related to the architecture of the habitat and these variations may deserve further study. In Cochabamba, the triatomines colonize rocky outcrops that provide stable and large weather-protected areas for mammals and triatomines, while in the study area, the habitat is more fragile. In fact, 1 year after the study, in May 2011, very few triatomines were captured (fewer than five specimens with around 30 traps); at the same time, we observed very strong erosion in the area, all the burrows being clogged with mud. The following years, 2012 and 2013, captures increased again, as did the number of new burrows. This shows the importance of local climate effects on mammalian habitats and therefore the consequences on triatomine populations (decline and/or dispersal).

### Spatial distribution and dispersal

In order to examine the wild population distribution in the study area, 50 sites corresponding to potential habitats of *T. infestans* were selected at the beginning of the study. The spatial analysis of these points showed an aggregated pattern for small distances of around 10 m. Among these sites, those where nymphs were captured showed a similar aggregation pattern. It is therefore understandable that the colonies of *T. infestans*, defined by the presence of nymphs, tend to be aggregated in this medium, similar to potential habitats. An alternative homogenous spatial distribution of the selected sites at the beginning of the study was not applied because a large proportion of the sites were positioned in places where it was impossible to hide a trap without the construction of an artificial cache; this would have created two categories of sites, one corresponding to potential habitats and the other playing only an attractive role, which would have complicated the study.

The natural dispersal of triatomines is assumed to be ensured by both walking (nymphs as well as adults) and flying (adults). Walking was only recently demonstrated for *T. infestans* nymphs and adults, showing this potential mode of dispersal between domestic and peridomestic structures in the arid Argentinean Chaco [23]. Flying is probably the most efficient mode of dispersal for adults and the reported flight distance for *T. infestans* is around 1 or 2 km, or more according to some reports [24]. However, the flight activity is scarce for triatomines and needs specific environmental and physiological conditions such as temperature, fasting, darkness, etc. [25]. Dispersal of wild *T. infestans* populations is poorly documented. Only one study conducted in the Cotapachi focus has detected restricted gene flow between close sylvatic sites, and the authors claimed that dispersal was mainly by walking [26].

The present study marked bugs for the first time, thus highlighting the displacement of nymphs and adults in their natural environment and measuring the distances covered. The third-, fourth-, and fifth-instar nymphs covered a few dozen meters and adults covered longer distances that at times exceeded 150 m, a sufficient distance to reach dwellings. Regarding nymphs, we must point out that marking is not preserved when they molt, leading to an underestimation when over time have passed after tagging. In agreement with the general idea that a minimal temperature is needed for adult flying, during the coldest months, adult displacements were rarely observed in this study. The difference between the males and females was more surprising regarding recaptures: while the proportions of recaptured specimens were similar for males (10/28 marked) and females (12/40 marked), the number of times females were recaptured was greater than for males. Moreover, no male was recaptured 2 months after marking, while one-third of female recaptures occurred after 2 months. This certainly shows differences in dispersal behavior between sexes: it may be that females preferentially engage in local displacements, whereas males may disperse further, which would explain the lower number of recaptured males in a small surface area. An alternative explanation would be that males die earlier than the females in nature, but no experimental result of the *T. infestans* developmental cycle supports this hypothesis. In addition, there is currently no argument to suggest that males are more susceptible to predators than females. In another context, we also observed different dispersal behaviors between sexes. This is the case of intradomicile incursions by *Meccus longipennis* reported in a village in the state of Nayarit (Mexico), where 76.3% of 76 collected adults were males. Since it was not possible to demonstrate peridomicile colonization in this village, these populations were more likely to have a sylvatic origin [27]. This suggests that males disperse greater distances than females and thus may play the main role in the colonization of new areas.

### Longevity in natural conditions

The survival and the resistance to fasting of the different triatomine stages is of epidemiological importance because they allow passive transportation, resistance to scarcity of feeding and to extreme climatic conditions, etc. Long survival times have been reported for experimentally breeding nymphs (several months) and adults (around 1 year), with great variations depending on the species. Interestingly, in the case of *Triatoma vitticeps*, Moreira and Spata [28] reported a surprising increase in survival of the different stages after only one blood meal [28]. In this work, marking the insects gave the first indications of longevity of nymphs and adults in natural conditions. Of the 92 recaptures of third-, fourth-, and fifth-instar nymphs, 14% were captured 3 months after marking and the longest time (more than 9 months) was observed for a fourth-instar nymph. This observation also suggests that it is not easy for the nymphs to find a food source that is sufficiently abundant to trigger molting, and their capacity to fast is probably an adaptive trait. The results also show that adults can live for a long time in nature: we recorded 2 months for males but even more for females, as high as 4.6, 7.7, and 10.6 months after marking. These data show that the longevity of *T. infestans* in the wild may be high, suggesting that this natural survival capacity would allow them to resist the scarcity of feeding hosts that is probably a very frequent trait of life in the wild. Moreover, this trait compensates the low rate of *T. infestans* generation per year (maximum two in warmer regions such as the Chaco) [22].

The dispersal of wild triatomine populations to the human habitat is a new challenge for the control of Chagas disease. This mechanism represents a risk of transmission of the disease that is emerging in several situations, even if the vectors are only passing visitors that do not colonize dwellings. It is therefore urgent to better understand the biology of these populations and the factors that are driving dispersal.

## Conclusions

The present study following Andean wild populations of *T. infestans* showed strong spatial and temporal stability during the year-long study. The capture-mark-recapture method was successful and allowed us to (i) demonstrate an adult dispersal time that heaviest during the rainy season, (ii) detect cases of nymph and adult longevity greater than 5 months, and (iii) demonstrate distances covered by nymphs of several dozen meters and more than 100 m by adults. The presence of a large wild population of *T. infestans*, also highly infected with *T. cruzi*, as previously shown [8], close to dwellings, still raises concern for the future and requires the establishment of a vigilance system.

## Competing interests

The authors declare that they have no competing interests.

## Authors’ contributions

Designed the study (PB, RB, EW, RB, CA, SFB), participated in field work (PB, RS, EW, RB, CA, CB, SD, SFB), computed data (RS, RB), operated the spatial information system (PB), performed spatial analysis (OD), analyzed data (RS,PB, SFB), wrote the manuscript (SFB, PB), contributed significantly to the corrections of the manuscript (EW). All authors read and approved the final version of the manuscript.
